# Synthesis of Mesoporous Silica Coated Gold Nanorods Loaded with Methylene Blue and Its Potentials in Antibacterial Applications

**DOI:** 10.3390/nano11051338

**Published:** 2021-05-19

**Authors:** Adrián Fernández-Lodeiro, Jamila Djafari, Javier Fernández-Lodeiro, Maria Paula Duarte, Elisabete Muchagato Mauricio, José Luis Capelo-Martínez, Carlos Lodeiro

**Affiliations:** 1 BIOSCOPE Research Group, LAQV-REQUIMTE, Chemistry Department, NOVA School of Science and Technology, FCT NOVA, NOVA University Lisbon, 2829-516 Lisbon, Portugal; a.lodeiro@fct.unl.pt (A.F.-L.); j.djafari@gmail.com (J.D.); jlcm@fct.unl.pt (J.L.C.-M.); 2 PROTEOMASS Scientific Society, Rua dos Inventores, Madam Parque, Caparica Campus, 2829-516 Almada, Portugal; 3 MEtRICs/DCTB/NOVA, School of Science and Technology, NOVA University Lisbon, Caparica Campus, 2829-516 Almada, Portugal; mpcd@fct.unl.pt; 4 CBIOS/DREAMS, Universidade Lusófona de Humanidades e Tecnologia, Campo Grande, 376, 1749-024 Lisboa, Portugal; p278@ulusofona.pt

**Keywords:** mesoporous silica, gold nanorod, methylene blue, antibacterial activity, gold nanoparticles

## Abstract

In this work, the successful preparation and characterization of gold nanorods (AuNRs) coated with a mesoporous silica shell (AuNRs@Simes) was achieved. Conjugation with methylene blue (MB) as a model drug using ultrasound-stimulated loading has been explored for further application in light-mediated antibacterial studies. Lyophilization of this conjugated nanosystem was analyzed using trehalose (TRH) as a cryogenic protector. The obtained stable dry formulation shows potent antimicrobial activity against Gram-negative (*Escherichia coli*) and Gram-positive (*Staphylococcus aureus*) bacteria after a simple post-treatment irradiation method with a red laser during a short time period.

## 1. Introduction

Since the pioneering works of Murphy and El-Sayed on synthesizing gold nanorods (AuNRs) using a wet chemical approach [1,2], a rising interest has been awakened in these fascinating nanostructures, mainly due to the intriguing optoelectronic properties that arise from their anisotropic nanoshape [3]. In AuNRs, two types of localized surface plasmon resonances (LSPR) are present: transversal (LSRPtrans) and longitudinal (LSRPlong), associated with the collective oscillations of conducted free electrons in the transverse and longitudinal directions of the gold rod, respectively [3,4].

The plasmon energy of the LSRPtrans is strongly linked with the aspect ratio (AR) in AuNRs—unlike gold spheres, which have similar plasmon energy within the 4–200 nm diameter range [3,4]. Consequently, in AuNRs, the LSRPtrans band can be shifted in an electromagnetic range from the middle visible to the NIR spectrum (600–1800 nm) through increases in AR [3,4].

Taking advantage of their optoelectronic properties, AuNRs can be implemented as an efficient photo-absorbing agent. Upon laser light irradiation, the efficient conversion of light into heat is achieved [5]. Such an effect can induce photothermal therapy (PTT) against cancer and trigger a release in drug-functionalized AuNRs [5,6,7]. Combined with photosensitizers (PS), they have been explored in photodynamic therapy (PDT) against cancer cells. [8,9] One of the main drawbacks of the direct use of AuNRs in biological applications is cetyltrimethylammonium bromide’s (CTAB) cytotoxicity [10,11]. In this sense, mesoporous silica can bring additional advantages. With the replacement of the CTAB bilayer with a SiO_2_ coating, cytotoxicity and non-specific interactions should be reduced and biological media stability should be improved. Furthermore, the mesoporous silica coating offers additional advantages, such as high pore volume and surface area, variable size, and biocompatibility. The porous nanostructured silica layer can act as molecular cargo for a drug delivery approach in medical applications [6,7,12,13].

Methylene blue (MB) is a cationic water-soluble photosensitizer (PS) that presents a high quantum yield of singlet oxygen (^1^O_2_) generation and has been considered as a photodynamic agent in different clinical applications [14,15]. MB has been used in photodynamic antimicrobial applications [16,17,18] as well as loading or grafting on silica-coated gold nanostructures (rods or pyramids), with applications in cancer cell photodynamic therapy (PDT) [9,19,20]. However, MB has the drawbacks of poor photostability and enzymatic degradation. To protect the MB against photodegradation and trigger the medium’s release upon laser irradiation, in this work, we have used an overlap with the LSRPlong band in AuNRs. It protects the MB molecules against photodegradation under the high absorption cross-section of the AuNRs [21]. Furthermore, under overlapping laser irradiation conditions with LSRPlong bands in AuNRs, an increase in the ^1^O_2_ generation has also been reported [20,21], and it is expected to improve the performance in photothermal processes [22,23].

Despite several works addressing the application of AuNR as antibacterial agents, most of them are limited to organic coatings [24,25,26,27]. However, according to our best knowledge, few works have explored the application of AuNRs@Simes nanostructures in antibacterial proposals [28].

In the present work, we present the synthesis of AuNRs coated with a mesoporous silica shell. The loading of AuNRs@Simes with MB was completed to explore its application as a photodynamic antibacterial material. The lyophilization of these conjugated nanosystems was investigated using trehalose as a cryogenic protector.

The antimicrobial properties of the obtained dry formulation, with and without red laser irradiation, have been investigated against Gram-negative (*E. coli*) and Gram-positive (*S. aureus*) bacteria. We have confirmed positive light-stimulated antimicrobial properties.

## 2. Materials and Methods

### 2.1. Materials 

Hexadecyltrimethylammonium bromide (CTAB), methylene blue (MB), sodium borohydride (NaBH_4_), D-(+)-trehalose dihydrate and tetraethyl orthosilicate (TEOS) were purchased from Sigma-Aldrich. Hydrogen tetrachloroaurate (III) trihydrate (HAuCl_4_ x 3H_2_O), potassium bromide (KBr) and silver nitrate (AgNO_3_) were purchased from Alfa Aesar. L(+)-ascorbic acid was purchased from Panreac. All reagents were used without further purification. The water was ultra-pure grade (type I), obtained with a Milli-Q Simplicity system. All spectroscopy and standard chemical techniques were used to characterize all NPs. ζ-potential analysis was conducted in a MALVERN model ZS instrument (Malvern Instruments Ltd., Malvern, UK, PROTEOMASS Scientific Society, BIOSCOPE facility). Ultraviolet–visible (UV–Vis) was conducted in a Jasco-650 spectrophotometer with temperature control (JASCO International, Tokyo, Japan, PROTEOMASS Scientific Society, BIOSCOPE facility). Fourier transform infrared (FT-IR) spectroscopy was performed using a Bruker Tensor 27 (Bruker Optik, GmbH, Berlin, Germany). Samples were prepared in KBr disks. Lyophilization was conducted in a CHRIST model Alpha 1-2 LDplus instrument (Martin Christ Gefriertrocknungsanlagen GmbH, Osterode, Germany, PROTEOMASS Scientific Society, BIOSCOPE facility). Transmission electron microscopy (TEM) analysis was performed using a TEM microscopy JEOL JEM1010 working at 100 kV to obtain low-magnification images (JEOL, Tokyo, Japan, University of Vigo, Vigo, Spain). The size of particles and dispersion histograms were calculated from TEM micrographs using the ImageJ package [29]. Scanning electron microscopy (SEM) analysis was performed using a Carl Zeiss AURIGA Crossbeam SEM-FIB microscope.

### 2.2. Synthesis of Gold Nanorods 

Briefly, a CTAB solution (10 mL, 0.1 M) was mixed with HAuCl_4_ (50 µL, 0.05 M) in a water bath at 30 °C. Then, an ice-cold, freshly prepared solution of NaBH_4_ (0.6 mL, 0.01 M) was rapidly injected. The brownish-yellow seed solution was stirred for 30 s and left undisturbed at 30 °C. The seed solution was used within 2 to 5 h. The growth solution consisted of a mixture of CTAB (80 mL, 0.1 M), HAuCl_4_ (4 mL, 0.01 M), AgNO_3_ (360 µL, 0.01 M), H_2_SO_4_ (1.6 mL, 0.5 M) and ascorbic acid (640 µL, 0.1 M). The growth was initiated after the addition of 192 µL of seeds, and the temperature was kept at 30 °C during the whole process. After 3 h, the reaction was centrifuged three times (7000 rpm × 15 min). The pellets were redispersed in CTAB 1 mM until a final volume of 100 mL.

### 2.3. Preparation of AuNRs@Simes 

Briefly, NaOH solution (0.1 M) was added to 50 mL of AuNRs@CTAB ((Au^0^) = 0.38 mM) solution [30] in a round bottom flask, to achieve a pH between 10.5–11. After 15 min of gentle stirring, 360 µL of 20% *v/v* TEOS in methanol was injected for six minutes (60 µL each minute). The AuNRs solution was gently stirred for 30 min and kept undisturbed for 20 h at room temperature.

After 20 h, both reactions were centrifuged (7000 rpm × 12 min), and the pellets dispersed in MeOH. Then the samples were centrifuged several times in MeOH at 6000 rpm for 12 min and finally redispersed in 10 mL of MeOH. After the first wash in MeOH, an aliquot was centrifuged repeatedly in water (denoted as AuNRs@Si-Water). The nanoparticles can be stored in MeOH, at 4 °C, which preserves all their properties for 12 months without degradation (data not shown).

### 2.4. Drug Loading 

Before proceeding with the incubation step, the AuNRs@Simes were successively washed with Milli-Q water (7000 rpm × 12 min) to remove all the MeOH. Finally, all samples were dispersed in 10 mL of Milli-Q water. To complete the loading, 1 mL of MB water solution (0.1 mg/mL) was added drop by drop to 1 mL of purified AuNRs@Simes. The mixture was incubated under stirring for 5 min, at room temperature, before immersion in a low-frequency ultrasonic bath, at 35 kHz for 1 min under manual stirring.. Then the solution was left for 10 min undisturbed and purified through centrifugation (7000 rpm × 15 min) in MQ water until the supernatant became clear. The AuNRs@Simes-MB were taken to a final volume of 1 mL of Milli-Q water. All the supernatants were collected, and the entrapment efficiency was determined from the relation [6,31]:

%Entrapment Efficiency = (Drug added-Free “unentrapped drug”)/(Drug added) × 100(1)

### 2.5. Freeze Drying of AuNRs@Simes-Drug 

To the AuNRs@Simes-MB solution, a water solution of trehalose (1 mL, 30 mM) was added to a final volume of 2 mL before immersing in liquid nitrogen for 5 min. The samples were lyophilized, and a blue powder was obtained. Empty AuNRs@Simes (without the addition of the drugs) were treated equally as a control. After resuspension of the lyophilized powder, the solution was centrifuged and resuspended in Milli-Q water to remove all the sugar shells to obtain the SEM images.

### 2.6. Antibacterial Activity

The antimicrobial activity was assayed against *E. coli* ATCC8739 (Gram-negative bacteria) and *S. aureus* ATCC6538 (Gram-positive bacteria). Glycerol stock cultures stored at −80 °C were inoculated in Tryptic Soy Agar (TSA) (Biokar, Allone, France) and incubated overnight at 35 ± 2 °C. Subsequently, isolated colonies were transferred to a 0.85% NaCl solution. The suspension’s turbidity was adjusted to 0.5 on the McFarland scale (McFarland densitometer, Model Den-1B, Grant Instruments, England), corresponding to 1 to 2 × 108 CFU/mL [32].

Samples (AuNRs@Simes, and AuNRs@Simes-MB) were dissolved in double-distilled water (2 mg/mL). MB solutions were also prepared in double-distilled water at a concentration of 0.015 mg/mL for MB. All solutions were prepared and handled under light-restricted conditions.

The antibacterial assays were performed according to the procedure described by Pérez-Laguna and co-workers with slight modifications [33]. The bacterial suspensions (0.5 McFarland) were deposited in 96-well microplates and mixed with the same volume of the different samples under study (AuNRS@Simes, AuNRS@Simes-MB and MB) or, in the case of the control, with double-distilled water. Microplates were prepared in duplicate, with one of them kept in the dark and the other irradiated with a red laser (JD-850, max output power 200 mW, wavelength 650 ± 10 nm), and at a distance of 2.5 cm from the top of each well, for 4 min. Then, the irradiated and non-irradiated bacterial suspensions were diluted in 0.85% NaCl (from 10–1 to 10–5), cultured on TSA and incubated overnight at 35 ± 2 °C. Viable bacteria, in colony-forming units (CFUs), were determined by colony counting in TSA plates containing between 30 and 300 colonies. All experiments were carried out at least three times.

### 2.7. Statistics in Bacterial Samples

As the assumptions of normality and homogeneity of variance (Cochran, Hartley and Bartlett tests) were verified, one-way analysis of variance (ANOVA) followed by Tukey’s test was used to identify significant differences between results. Statistical analyses were tested at the 0.05 level of probability with the software STATISTICA™ 7.0 (StatSoft Tulsa, OK, USA).

## 3. Results and Discussion

### 3.1. Synthesis of Gold Nanorods

**Figure 1:**
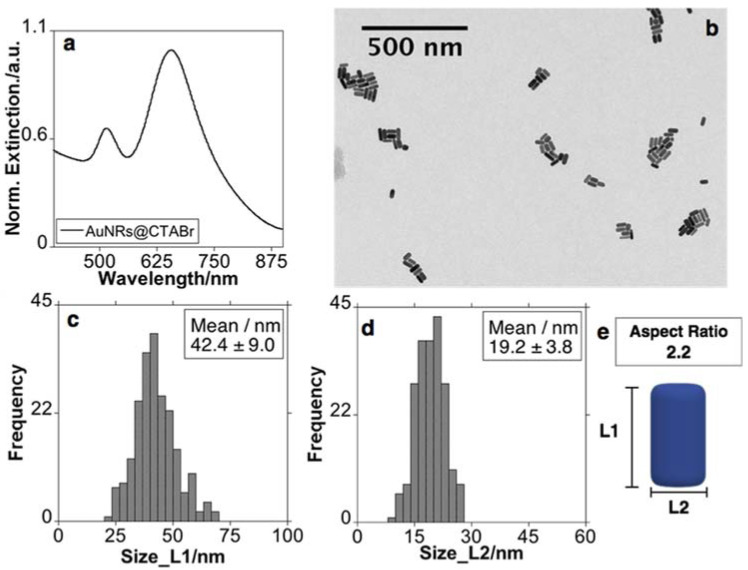
Normalized extinction spectra (**a**), representative TEM images (**b**), histograms (counting of 200 nanoparticles), and graphic representation of AuNRs obtained (**c**–**e**). The colloid solution was washed using CTAB solution of 1 mM before use as a precursor in the subsequent deposition silica process.

The AuNRs were synthesized according to a previously reported seed-mediated silver ion-assisted methodology, using CTAB as a template, with some minor modifications [7]. As shown in Figure 1a, the prepared sample presented two absorption bands, one weak band at ca. 518 nm and a strong band centered on ca. 656 nm assigned to the transverse (LSPRtrans) and longitudinal (LSPRlong) plasmon band, respectively. The TEM images showed a colloidal solution essentially composed of rod-shaped gold nanoparticles with a length of 42.4 ± 9.0 nm and a width of 19.2 ± 3.8 nm. With these dimensions, the AuNRs presented an aspect ratio (AR) of ≈ 2.2 (Figure 1).

### 3.2. Synthesis and Purification of AuNRs@Simes

**Figure 2:**
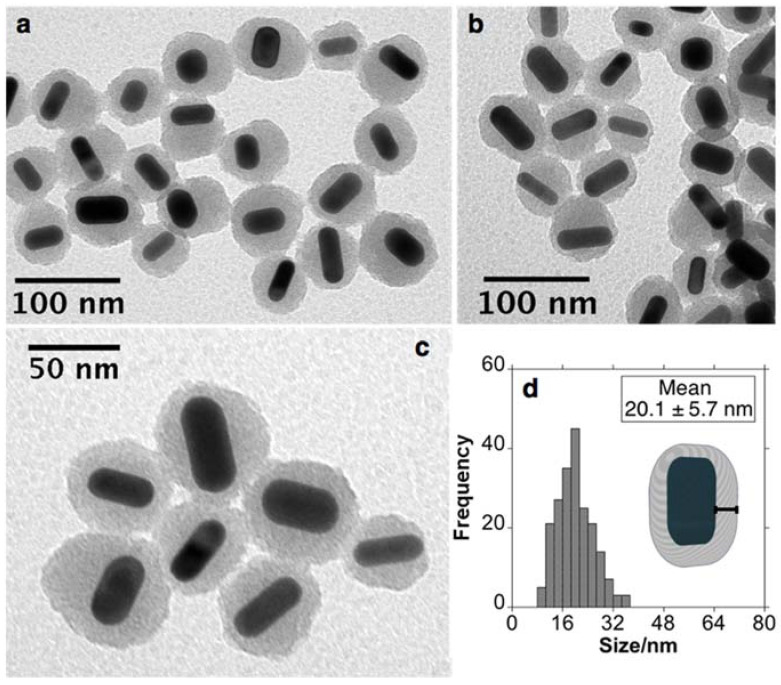
Representative TEM images (**a**–**c**), histograms and graphic representation of AuNRs@Simes (**d**).

The mesoporous silica coating was achieved using a modified Stöber method reported by Tracy et al. with some modifications [34]. The controlled deposition of mesoporous silica was completed using TEOS as silica precursor in a basic medium (pH ≈ 10.5–11). The presence of CTAB serves as a template to obtain mesostructured silica growth. After silica deposition, AuNRs@Simes presented a silica thickness of 20.1 ± 5.7 nm. More important, core-free silica NPs were not detected in the TEM images (see Figure 2).

**Figure 3:**
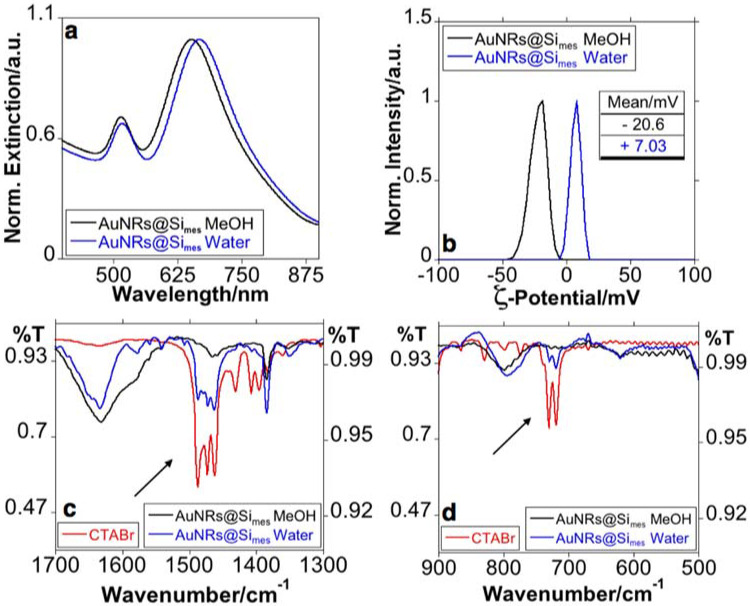
Normalized extinction spectra (**a**), z-potential (**b**) and FT-IR extensions spectra between 1700 and 1300 cm^–1^ (**c**) and 900 and 500 cm^−1^ (**d**) of AuNRs@Simes purified with MeOH or water. (The UV-Vis spectra and z-potential were recorded in Milli-Q water upon the corresponding purification process).

To eliminate the CTAB, the AuNRs were subjected to a purification process through successive centrifugations (see experimental section). The sample’s purification using water resulted in a red-shift of the LSPR to ca. 667 nm (Figure 3a). Conversely, upon successive MeOH and then water washes, the LSPR of the colloid solution resulted in a blue-shift to 653 nm, bringing the LSPR near to that of AuNRs without silica shell (Figure 3a). This behavior has recently been conveniently explained by J. B. Tracy and co-workers. The authors attributed the blue-shift to the dissolution of CTAB molecules that remain in the template in the mesostructured silica shell [35]. The ζ-potential of the sample purified in water was positive (+7 mV). In contrast, in the opposite case, the MeOH purification showed negative values (−20 mV) (see Figure 3b).

We have investigated the chemical composition of the AuNRs@Simes after purification with water or MeOH using FT-IR spectroscopy (Figure 3c,d).

The CTAB spectra clearly showed the typical vibrational modes previously reported in the literature associated with the −CH_2_, −CH_3_ or [−N(CH_3_)_3_]^+^ groups that form the molecule (located between 1481–1437 and 719–730 cm^−1^, see Figure 3c,d) [36,37,38,39]. Deformational vibrations of adsorbed water molecules give rise to the bands located at 1634 cm^−1^ [40,41]. More important, the CTAB groups’ signals are only evident in the spectrum obtained from the sample purified in water (Figure 3c,d).

The blue-shift in the LSPR agreed with previous reports [35,42], while the negative z-potential, and the marked decrease in the signals produced by CTAB groups in FT-IR analysis for AuNRs@Simes purified in MeOH suggest a substantial CTAB removal.

Then, AuNRs@Simes were used to complete the loading experiments using methylene blue as a model cationic drug. The colloidal solutions were gently washed in water to remove any remaining MeOH before the conjugation.

### 3.3. Drug Loaded and Freeze-Drying Experiments

**Figure 4:**
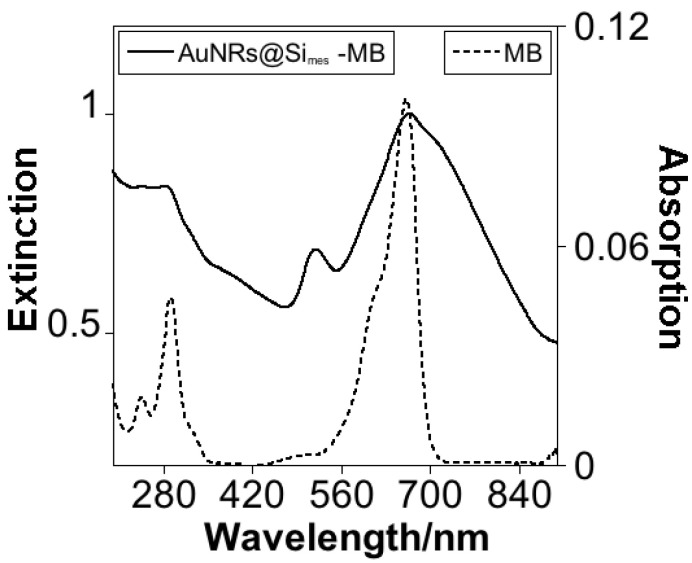
Absorption spectra of an aliquot of AuNRs@Simes-MB (axis y—left) and MB (axis y—right) in water solution.

We further investigated the drug loading capacity, exploring the encapsulation of MB using an ultrasound-assisted technique for 15 min. The ultrasound irradiation was used to avoid the aggregation of the nanomaterials during the incubation process due to the difference in charge of the AuNRs and the MB [43]. The entrapment efficiency was calculated by analyzing the UV-Vis spectra of supernatants from the calibration curve yielding 13.6 ± 3.2% encapsulated MB. After the loading and purification process, the LSPR showed a red-shift to 668 nm (Figure 4). It can be explained by the sensitivity of the AuNRs to the medium’s refractive index, in agreement with previous reports, where the LSPR also were red-shifted after the internalization of MB [20].

To avoid the diffusion of an entrapped drug in the solution during storage periods, AuNRs@Simes-MB was subject to lyophilization, applying freeze-dry techniques. Sugars, specifically trehalose, are an excellent candidate to lyophilize pharmaceutical products due to the absence of internal hydrogen bonds, lower hygroscopicity, lower chemical reactivity, and higher glass transition temperature (Tg) [44,45,46,47]. Nanoparticles encased in high Tg amorphous carbohydrates allow us to store the products at room temperature. Trehalose was stated as the best cryoprotectant for lyophilizing mesoporous silica nanoparticles when compared with other common cryoprotectants, such as mannitol or sorbitol. The presence of trehalose can prevent aggregation and allow an easy reconstruction, maintaining the same size and polydispersity index (PDI) compared to the prior wet formulation. [48,49]

**Figure 5:**
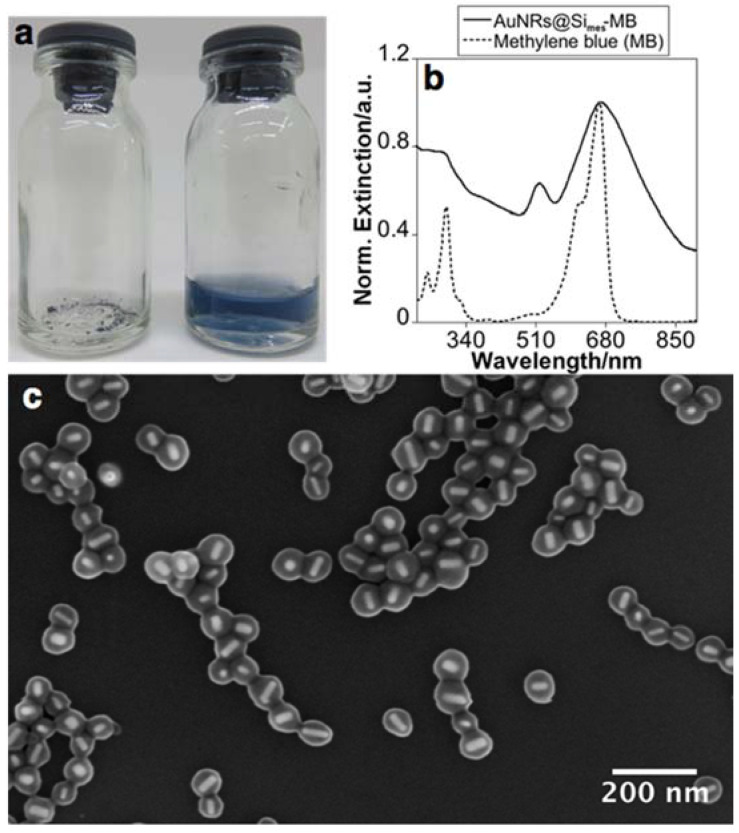
Lyophilized and resuspended sample (**a**), normalized extinction spectra of AuNRs@Simes-MB after resuspension and MB in water solution (**b**) and representative SEM images after resuspension in Milli-Q water (**c**).

After the lyophilization process, a powder was obtained (Figure 5a). The powders facilitated easy redispersion in water without applying external inputs (Figure 5a). As revealed by UV-Vis spectra and SEM images, the optical and structural features were preserved during the lyophilization process (Figure 5b,c). They maintain their properties for at least 12 months after lyophilization and storage at room temperature.

### 3.4. Exploring the Application of AuNRs@Simes-MB

Bacteria can be classified into Gram-negative and Gram-positive based on their cell wall composition. Gram-positive bacteria contain a thicker peptidoglycan layer with teichoic wall acids and lipoteichoic acids covalently attached; Gram-negative bacteria have a thinner peptidoglycan layer surrounded by an outer membrane. The outer membrane is a lipid bilayer, where the inner leaflet is composed of phospholipids and the outer leaflet is composed of highly negatively charged lipopolysaccharides (LPS) [50,51]. Gram-negative bacteria tend to be more resistant to antimicrobial agents than Gram-positive bacteria because of the additional protection afforded by the outer membrane.

Based on these differences in composition and sensitivity, the antibacterial activity of the lyophilized AuNRs@Simes formulation was assayed against Gram-negative (*E. coli*) and Gram-positive bacteria (*S. aureus*) (Table 1). Neither *E. coli* nor *S. aureus* were affected by laser irradiation as no significant differences have been observed in the number of CFU/mL between irradiated and non-irradiated controls. The efficiency of AuNRs as photothermal agents strongly depends on the absorbance band’s position and the aspect ratio of the sample. In this case, the selected AuNRs with a band at 650 nm overlapping the MB show no PTT effects alone, being biocompatible even after laser irradiation. Even if the negligible result of the AuNRs alone was unexpected, it indicates an efficient protocol for the removal of the CTAB template or remaining MeOH after purification, which can produce toxicity and lead to a misinterpretation of the results [24].

Regarding AuNRs@Simes-MB and MB, the results showed that both samples’ antibacterial activity was strongly affected by the exposure to the red laser. Accordingly, after irradiation, both MB and AuNRs@Simes-MB showed a pronounced antibacterial activity (~8 log10 unit reduction) against *E. coli* and *S. aureus*. More important, MB without laser exposure reduced the number of CFU/mL of *E. coli* in about 2 log10 cycles, an effect that was not observed with AuNRs@Simes-MB without laser (Table 1).

We have tested the MB effect alone and in combination with trehalose, irradiated and non-irradiated with the laser. The presence of trehalose can enhance bacterial survival in the *E. coli* sample without laser irradiation (in about 1 log10 cycle). It can be explained as due to trehalose’s presence, which can significantly decrease the toxicity effect, as stated in other systems [52,53,54,55]. Despite this, with trehalose’s presence there is still some observed toxicity on the *E. coli* bacteria, toxicity which is prevented in the AuNRs@Simes-MB sample.

The results obtained suggest that *E. coli* has a higher sensitivity to MB than *S. aureus*. Positively charged photosensitizers are expected to be more effective bacterial inactivators than neutral molecules against Gram-negative bacteria, whose outer membranes are composed of negatively charged lipopolysaccharides. In contrast, Gram-positive bacteria may be inactivated more effectively by neutral or negatively charged agents [56].

**Figure 6:**
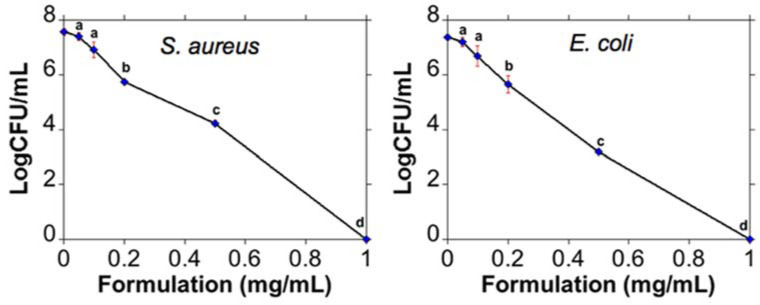
Inactivation of *S. aureus* (**left)** and *E. coli* (**right**) using different concentrations of formulation (AuNRs@Simes-MB) after exposure to the red laser. Other letters within the same curve indicate statistically significant differences among Log10 CFU/mL (*p* < 0.05).

According to the above, under the same experimental conditions, *E. coli* proved to be more sensitive to the bactericidal effects of AuNRs@Simes-MB than *S. aureus* (Figure 6) as a more pronounced log10 unit decrease was achieved with the same concentration of AuNRs@Simes-MB (3 and 4 log10 unit decrease with 0.5 mg/mL for *S. aureus* and *E. coli*, respectively).

## 4. Conclusions

We have successfully synthesized AuNRs@CTAB in an aqueous solution matching the photosensitiser absorption band. The controlled deposition of mesoporous silica coating (≈20 nm) on AuNRs was successfully realized, and an efficient removal of the cytotoxic CTAB has been performed. Conjugation with MB was obtained using ultrasound techniques in a short time period. The subsequent lyophilization in the presence of trehalose as a cryoprotection molecule was applied successfully, giving a long shelf-life to the system. We have observed that there were no appreciable changes in the optical or nanostructuration features after redispersion. The formulation (AuNRs@Simes-MB) obtained was effective against Gram-positive and Gram-negative bacteria after laser irradiation. The lack of toxicity in the formulation system without the laser exposure, even with the presence of MB and the toxicity after the laser exposure, indicates that it can be a promising system with remote light-triggered drug delivery properties.

A full assessment of the AuNRs system should be conducted to determine their efficacy in vitro and their toxicity, compared with the more conventional photodynamic agents. This work has the potential to aid in the development of a more effective PDT for antibacterial applications.

## Figures and Tables

**Table 1:** Antibacterial activity of AuNRs samples against *E. coli* and *S. aureus*. Different letters within the same column indicate statistically significant differences among samples (*p* < 0.05).

Samples	*E. coli*		*S. aureus*	
	Log_10_CFU/mL	Log Reduction	Log_10_CFU/mL	Log Reduction
I Control	7.58 ± 0.20 (a)	-	7.55 ± 0.11 (a, b)	-
NR Control	7.70 ± 0.24 (a)	-	7.51 ± 0.06 (a, b, c)	-
* AuNRs@Simes (1 mg/mL)	7.88 ± 0.03 (a)	<1	6.70 ± 0.26 (e)	<1
** AuNRs@Simes (1 mg/mL)	7.98 ± 0.09 (a)	<1	6.97 ± 0.13 (d, e)	<1
* AuNRs@Simes-MB (1 mg/mL)	<1	>7.58 ± 0.00	<1	>7.55 ± 0.00
** AuNRs@Simes-MB (1 mg/mL)	7.66 ± 0.10 (a)	<1	7.13 ± 0.15 (c, d)	<1
* MB (0.015 mg/mL)	<1	>7.58 ± 0.00	<1	>7.55 ± 0.00
** MB (0.015 mg/mL)	5.52 ± 0.21 (c)	2.18 ± 0.21	7.20 ± 0.12 (b, c, d)	<1
* TRH (10.34 mg/mL)	7.86 ± 0.13 (a)	<1	7.95 ± 0.01 (a)	<1
* MB (0.015 mg/mL) + TRH (10 mg/mL)	<1	>7.58 ± 0.00	<1	>7.55 ± 0.00
** MB (0.015 mg/mL) + TRH (10 mg/mL)	6.64 ± 0.07 (b)	1.06 ± 0.07	7.20 ± 0.16 (b, c, d)	<1

* I = Irradiated; ** NR = Non-irradiated; TRH = Trehalose; MB = Methylene Blue.

## Data Availability

Not applicable.
